# Posterior transdural resection of giant calcified thoracic disc herniation in a case series of 12 patients

**DOI:** 10.1007/s10143-020-01413-0

**Published:** 2020-10-16

**Authors:** Chiara Negwer, Vicki M. Butenschoen, Sandro M. Krieg, Bernhard Meyer

**Affiliations:** Department of Neurosurgery, School of Medicine, Technical University of Munich, Klinikum rechts der Isar, Ismaninger Str 22, 81675 Munich, Germany

**Keywords:** Transdural surgery, Giant thoracic disc herniation

## Abstract

Calcified thoracic disc herniations present a rare and challenging entity. Due to the close proximity to the spinal cord and relative narrowing of the spinal canal, the optimal approach remains a matter of debate. While the transthoracic approach is usually preferred, we adapted a new technique described in 2012: the transdural posterior approach. Our aim was to evaluate its benefits in patients with giant thoracic disc protrusions. We retrospectively reviewed all patients treated in our neurosurgical department from July 2012 to March 2020. Demographics, pre- and postoperative clinical status, and operative technique and complications were extracted and analyzed. In total, 12 patients underwent a posterior transdural resection of giant calcified thoracic hard discs between 2012 and 2020. All patients underwent a posterior decompression (laminectomy, hemilaminectomy, or laminoplasty). The median duration of surgery was 152 min. Transient postoperative neurological deterioration occurred in 4 patients, with complete recovery until time of discharge. No patient underwent a surgical revision. The transdural resection of giant calcified thoracic hard discs through a posterior approach provides an excellent decompression with sufficient visualization of the spinal cord and a satisfying postoperative outcome.

**Electronic supplementary material:**

The online version of this article (10.1007/s10143-020-01413-0) contains supplementary material, which is available to authorized users.

## Introduction

Thoracic disc herniations remain an uncommon cause for compression of the spinal cord and symptoms of myelopathy [1–4]. Compared with cervical and lumbar disc herniations, the calcification rate has been reported to be 30–70% [5], which contributes to the complexity of surgery removal. Several surgical approaches have been proposed to perform a resection in symptomatic patients, including the transthoracic approach [6–8], posterior costotransversectomy [9], and transpedicular approaches [10]. The optimal treatment remains a matter of debate [11, 12], and complications such as spinal instability, cerebrospinal fluid (CSF) leakage, and pleural fistulas are described [13]. In 2012, Coppes et al. introduced a case series of 13 patients operated on, with a novel technique for central thoracic disc herniation removal, using a posterior transdural approach for central disc herniations of the thoracic spine [14]. While the technique has been described before in smaller sample sizes [5], numerous advantages were described, including a low blood loss and perioperative morbidity, and a smaller invasiveness without the need for chest tube placement. Most patients treated in this series suffered from non-calcified thoracic disc herniations with moderate size, so the feasibility and outcome of patients with giant thoracic hard discs has not been examined yet.

We hereby aim to evaluate the feasibility and neurological outcome of patients suffering from giant calcified central disc herniations (occupying more than 40% of the spinal canal according to Hott et al. [3]) and symptoms of myelopathy, treated through a posterior transdural approach.

## Methods

### Study cohort

We performed a retrospective cohort study of all consecutive patients treated in our neurosurgical department for giant thoracic herniation and compression of the spinal cord, with symptoms of myelopathy from July 2012 to February 2020. Inclusion criteria were giant thoracic disc herniation, surgical treatment with a transdural posterior approach for the resection of the disc herniation, complete pre- and postoperative documentation of the surgical approach, and neurological and clinical status.

### Study design

We conducted a retrospective monocentric analysis in a high-volume neurosurgical center. Relevant details leading to the indication of surgery, intraoperative findings and procedures, and postoperative radiographic and clinical outcomes were recorded. Clinical status was assessed using the modified Japanese Orthopedic Association Score (mJOA), the Medical Research Council grading for muscle strength (MRC), and American Spinal Injury Association (ASIA) Scale.

### Ethics

The presented study meets the ethical standards outlined in the Declaration of Helsinki, ethics approval was obtained, and the positive vote was registered under the number 159/16. Patient consent was obtained for publication of the operative video.

### Statistics

Statistical analyses were performed using SPSS Statistics Version 26.0.0 (IBM, Chicago, IL). Binomial dichotomized data were compared using Fisher’s exact test and categorical data were compared using the chi-squared test.

Median or mean values were compared using a Student’s *t* test when appropriate. The association between potential factors and transient and permanent postoperative impairment (using follow-up data or discharge data for those with a missing follow-up) was analyzed using ANOVA for variance testing and for linear regression modeling. The following factors were assumed as potentially predictive: surgery duration, preoperative treatment, and age.

*P* values less than .05 were considered statistically significant.

## Results

### Patient population

In total, 12 patients were included for analyses. Four patients were male (33.3%) and 8 patients female (66.7%). Median age was 56 years (range 28 to 94 years, standard deviation SD ± 22 years). All patients suffered from giant calcified thoracic disc protrusions. Previous surgical treatment of the thoracic segment has been performed in 4 cases (33.3%) with/or complicative and unsatisfying operative results through a transthoracic approach (*n* = 1), hemilaminectomy (*n* = 2), and laminectomy (*n* = 1). Two of the 4 patients experienced a neurological deterioration after the first surgery—imaging showed a residual compression (*n* = 1) or postoperative hemorrhage (*n* = 1) with the need for further surgical treatment. In 2 patients, the neurological deficits remained stable but postoperative imaging revealed a persistent compression of the spinal cord. All patients were transferred from foreign hospitals.

**Fig. 1:**
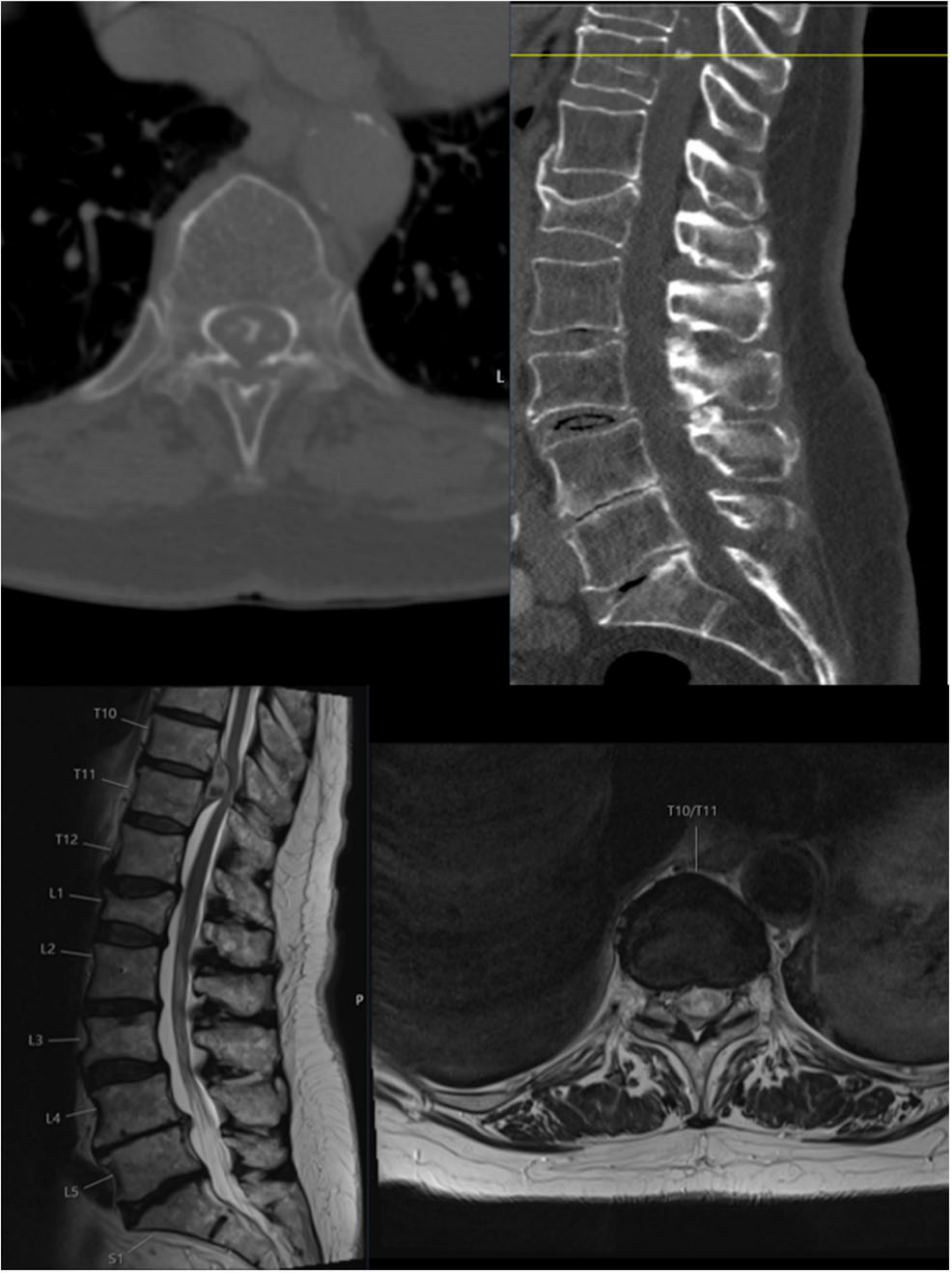
Preoperative CT (upper image) and MRI (lower image) scan of a patient suffering from a calcified giant disc herniation at the level Th10/11 with axial and sagittal plane showing the calcification (➔) and compression of the spinal cord

All patients underwent a preoperative MRI and CT scan to estimate the calcification of the giant extradural disc herniation (Fig. 1). Locations of the thoracic disc herniation were in descending order: Th8/9 in 3 cases, Th5/6 (2), Th9/10 (2), Th10/11 (2), Th7/8 (1), Th10/11 (1), and Th11/12 (1).

Median symptoms duration was 7 months, ranging from 2 weeks to 2 years. All patients presented with symptoms of myelopathy (ASIA D), one patient was paraplegic prior to surgery (ASIA B). Mean and median preoperative mJOA score was 15 and 16 out of 18 points. Most patients suffered from gait disturbance (median mJOA lower limb motor score 5/7). In total, 8/12 patients (66.7%) suffered from a mild myelopathy (mJOA 15–17), 3 patients from a moderate myelopathy (25%, mJOA 12–14), and 1 patient from a severe myelopathy (mJOA of 9 points).

Four patients (33.3%) complained of preoperative vegetative bladder dysfunction. Hypoesthesia of the lower extremities was described by 8 patients (66.7%). Motor deficits of the lower extremity were present in 4 patients (33.3%, MRC range 2–4/5) (Table 1).

**Table 1:** Patient demographics with sex, age, level of surgery, approach (*LE* laminectomy, *HL* hemilaminectomy, *LP* laminoplasty), presence of myelopathy, hypoesthesia, bladder dysfunction, and motor deficits

ID	Sex	Age	Level	Previous surgery	Approach	Myelopathy	Hypoesthesia	Bladder dysfunction	Motor deficit
1	Female	74	Th 9/10	Yes	LE	Yes	Yes	Yes	Yes
2	Male	70	Th 11/12	Yes	HL	Yes	Yes	Yes	No
3	Male	28	Th 10/11	Yes	HL	Yes	Yes	No	No
4	Male	55	Th 8/9	No	HL	Yes	No	No	No
5	Female	94	Th 8/9	No	LE	Yes	Yes	Yes	No
6	Female	45	Th 12/L1	No	LE	Yes	No	No	No
7	Female	59	Th 8/9	No	LE	Yes	No	No	No
8	Male	33	Th 7/8	No	LP	Yes	Yes	Yes	Yes
9	Female	38	Th 5/6	Yes	LE	Yes	Yes	No	Yes
10	Female	83	Th 10/11	No	LE	Yes	Yes	No	No
11	Female	36	Th 9/10	No	LP	Yes	No	No	Yes
12	Female	74	Th 5/6	No	LP	Yes	Yes	No	No

### Surgical procedure

All patients were placed in a prone position. Fluoroscopic control of the operated level was performed prior to the skin incision. Preoperative prophylaxis with an intravenous antibiotic was administered in all patients.

**Fig. 2:**
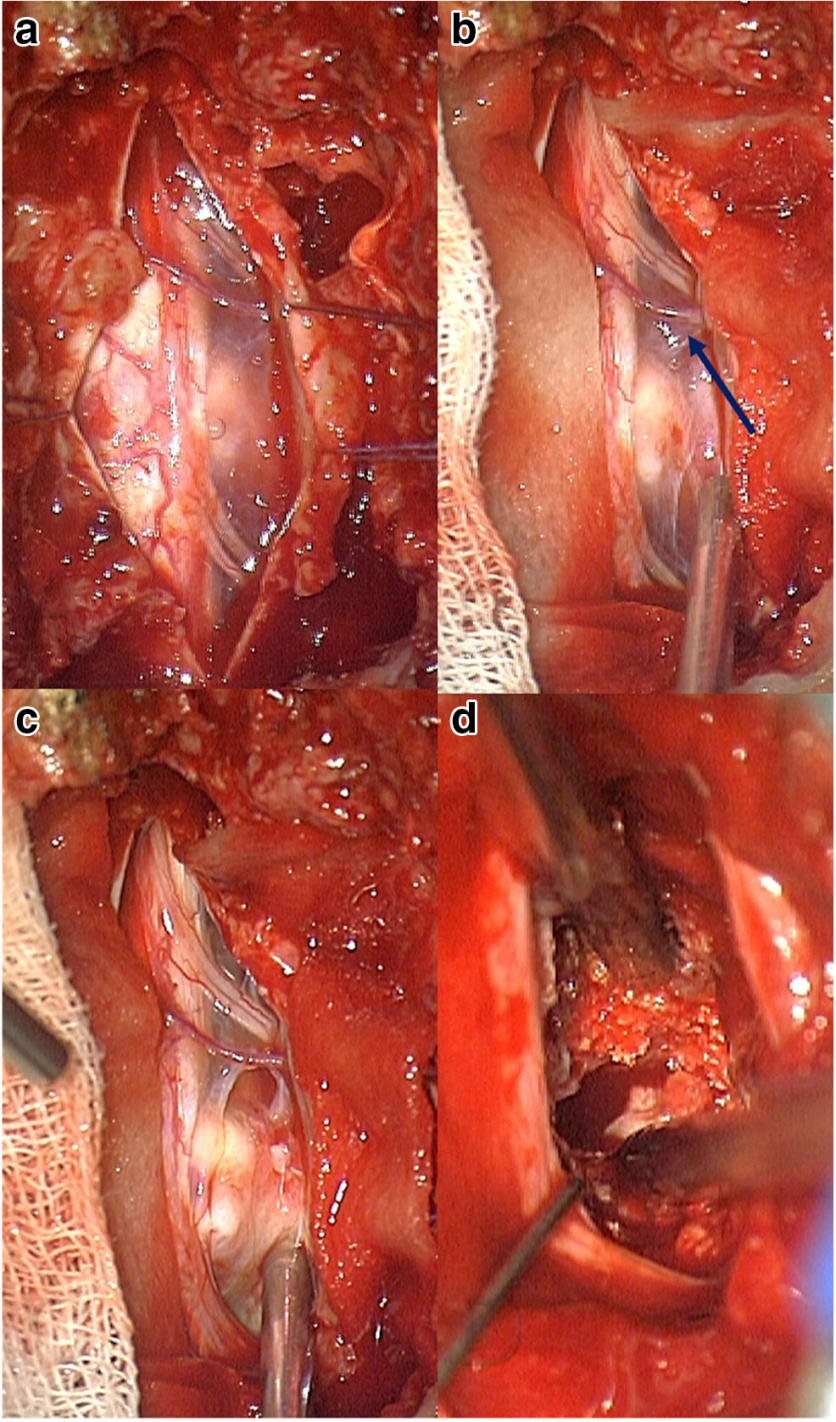
Intraoperative photograph of the spinal cord and the giant thoracic disc prolaps after durotomy (**a**). The denticulate ligament is sutured to medialize the spinal cord (**b**). The ventral dura is opened to access the disc prolaps (**c**). Finally the calcified mass is debulked (**d**)

First, we performed a midline incision and identified the spinous process, followed by subperiosteal preparation of the paraspinal musculature exposing the (hemi)-lamina. A (hemi)-laminectomy was performed in order to perform a (para)median durotomy. The spinal cord is slightly and carefully medialized using a Gore-Tex® suture to the denticulate ligament achieving so a dorsiflexion and rotation of the spinal cord. The ventral dura is opened to provide a direct view on the calcified disc mass which is subsequently resected. For the resection, we used in addition to forceps a CUSA and a high-speed drill for debulking the calcified mass (Fig. 2). Dural closure is performed anteriorly with an inlay-outlay fibrin sealant patch and dorsally with a running Gore-Tex® suture. In 6 patients, we opted for a laminectomy (50%), 3 patients underwent a laminoplasty (25%), and 3 patients a hemilaminectomy (25%). The approach was chosen at the discretion of the surgeon.

Median duration of surgery was 152 (± 71.4) minutes (range 83–289 min); intraoperative monitoring (IONM) with evoked potentials (motor MEP and somatosensory SSEP) was performed in 8 patients (66.7%) with stable MEPs in 75% of the cases and two patients with intraoperative drop of MEP amplitude but complete recovery during surgery. The use of IONM was depending on the individual decision made by the treating surgeon. We did not place any drain or restrict the patient to bed rest.

### Outcome

Transient neurological deterioration was observed in 4 patients (33.3%) with accentuated gait disturbance in one patient and decreased motor function (up to MRC 4/5 in one patient and MRC 3/5 in two patients). All patients recovered to the preoperative status during the same hospital stay until discharge. Median length of hospital stay was 10 days (± 5.1) (range 3 to 18 days). Mean and median postoperative mJOA score before discharge was 15 and 16 points (identical to the preoperative mJOA score). Eight out of 12 patients were discharged with a stable mJOA score (66.7%), 2 patients improved (1 point and 2 points), and 2 patients worsened (both remaining with a mild myelopathy).

Patient follow-up was obtained in 9 out of 12 patients (75%). Median follow-up was 303 days (range 77 to 2429 days). At follow-up, six patients reported an improvement of the clinical function compared with the initial preoperative status (66.7%) and three patients described a stable clinical status (33.3%). None of the patients experienced a secondary neurological deterioration or a need for further surgical treatment. Of the 4 patients experiencing a direct postoperative transient deterioration, 3 were examined at follow-up: 2 patients reported on an improvement of neurological symptoms compared with the preoperative status, one patient remained stable. Mean and median mJOA score was 16 and 17 points.

### Complications

In total, complications occurred in six patients (50%), including minor adverse events such as urinary tract infection (16.7%), and postoperative transient neurological deterioration (33.3%) which resolved until discharge. MRI and CAT scans were performed in all patients. We did not encounter any cerebrospinal fluid (CSF) leakage or postoperative hemorrhage so no patient underwent a surgical revision (Table 2).

**Table 2:** Complications and postoperative outcome at follow-up with number of cases (*n*) and percentage (%), *UTI* urinary tract infection

	*n*	%
Complications		
CSF leakage	0/12	0
Neurological deterioration	4/12	33.3
Surgical revision	0/12	0
UTI	2/12	16.7
Follow-up	9/12	75
Improved	6/9	66.7
Stable	3/9	33.3
Worsened	0/9	0

#### Case presentation

An 83-year-old male patient presents in our outpatient department with symptoms of progressive gait disturbances and hypoesthesia of both lower extremities. The preoperative MRI and CT scan reveals an extradural intraspinal mass at the level Th10/11 and the patient was advised to undergo surgical removal (Fig. 1). Surgery was performed through a posterior transdural approach via a laminectomy Th10. Successful resection of the herniation was performed and the patient was discharged on postoperative day 8 after an uneventful clinical course.

#### Operative video

A 32-year-old male patient presented in our department with progressive paraparesis (ASIA D), bladder dysfunction, and genital hypesthesia. The MRI and CT scans revealed a giant hard disc in Th7/8 with compression of the spinal cord. Giant hard discs usually are surgically removed using a transthoracic approach; in this case, we opted for a less invasive posterior transdural approach. A laminoplasty Th7/8 was performed, and the giant hard disc could be completely removed with stable intraoperative monitoring (MEPs). Postoperative the patient had a transient deterioration of his neurological status, which improved until his discharge on the 12th postoperative day. This surgical video shows the safe application of the posterior transdural approach as an alternative surgical strategy to the transthoracic procedure.

## Discussion

In our case series of 12 patients with giant calcified disc herniations, resection through a posterior transdural approach was feasible in all patients, without a need for surgical revision.

### Neurological outcome and complications

While resection of the giant disc herniation was feasible in all patients, we observed a considerably high percentage of patients with transient minor neurological deterioration after surgery and complete recovery until discharge (4 patients, 33.3%). Compared with other approaches for thoracic disc herniations, this number seems rather high, but we need to highlight the fact that only patients with giant calcified disc herniation were included in this study: a patient group that usually is at much higher risk for postoperative neurological deterioration (3 to 25% with permanent neurological worsening after transthoracic surgery [3, 7, 15]). Furthermore, all patients in our cohort recovered from their neurological deterioration during the same hospital stay, leaving us with a 0% rate of a new permanent neurological deficit. Although the occurrence of neurological transient deterioration was higher, the mean LOH was comparable with patients treated with the transthoracic approach (11 days [16]). We observed either an arrested progression of myelopathy (33.3%) or improving clinical status (66.7%) within the follow-up.

In our cohort study, we did not encounter any postoperative CSF leakage with a need for surgical revision. Compared with published literature, CSF leakage rates range from 4 to 8% [14] with the transdural and 15% with the transthoracic approach [16].

### Surgical technique

While the transdural technique described in 2010 by Moon et al. used a bilateral approach through a laminectomy [5], Coppes et al. presented a unilateral approach performing a hemilaminectomy [14]. In our department, we decide individually upon the extent of lamina resection, and perform either a hemilaminectomy, laminectomy, or laminoplasty. We did not identify a difference in the relative risk for neurological deterioration depending on the used approach. On follow-up, no patient suffered from signs of segmental instability. Median surgery duration was 152 (± 71.4) minutes (range 83–289 min). Compared with the transthoracic approach, the duration of the transdural posterior approach is much shorter (mean duration in transthoracic surgery of giant thoracic disc herniation ranging from 251 min [17] to 344 min [16]). In July 2020, Shedid et al. [18] published a case series of 8 patients undergoing a posterolateral microscopic transpedicular approach with a tubular retractor for giant thoracic disc herniations. In total, 3/8 patients required a posterior fixation in order to achieve sufficient decompression to visualize the disc herniation. In our case series, none of the patients underwent a posterior fixation.

### Indication for the transdural approach

Reviewing the literature on thoracic disc herniations, authors seem to agree that giant central disc herniation should be treated with a good visibility and a minimal amount of manipulation of the spinal cord [3]. One advantage of the posterior transdural approach has already been mentioned by Coppes et al. and describes the possibility to remove central and lateral disc herniations or even meningiomas in cases of preoperative doubt on the mass’ entity. Due to the possibility to drain CSF, the transdural approach allows us to gain more operative space, hence less manipulation of the spinal cord.

In our cohort, four patients underwent a prior unsuccessful operative treatment through a posterior extradural (75%) or transthoracic (25%) approach. All patients were then successfully treated with the transdural approach at our department with satisfying clinical outcome at discharge.

### Limitations

We performed a retrospective analysis of all consecutive patients treated with the transdural approach for giant disc thoracic herniations.

The small sample size of 12 patients reflects the rarity of giant thoracic disc herniations. It limits the statistical power of analyses and potential correlations could be underrepresented.

## Conclusion

In our clinical case series comprising 12 patients with giant calcified thoracic disc herniations, all patients could successfully be treated with a posterior transdural approach. Overall, this approach showed a comparable safety to the alternative transthoracic procedure, hence a shorter time of surgery, quicker recovery due to its less invasive nature, and no need for additional instrumentation.

## Electronic supplementary material

ESM1 Operative video (MOV 749 mb)

## Data Availability

The datasets used and/or analyzed during the current study available from the corresponding author on reasonable request.
